# Consumption of low-fat dairy foods for 6 months improves insulin resistance without adversely affecting lipids or bodyweight in healthy adults: a randomized free-living cross-over study

**DOI:** 10.1186/1475-2891-12-56

**Published:** 2013-05-02

**Authors:** Todd C Rideout, Christopher P F Marinangeli, Heather Martin, Richard W Browne, Curtis B Rempel

**Affiliations:** 1 Department of Exercise and Nutrition Sciences, School of Public Health and Health Professions, University at Buffalo, Buffalo, New York, 14214, USA; 2 The Richardson Centre for Functional Foods and Nutraceuticals, University of Manitoba, Winnipeg, Manitoba, R3T 2N2, Canada; 3 Biotechnical and Clinical Laboratory Sciences, University at Buffalo, Buffalo, NY, 14214, USA

**Keywords:** Dairy, Glucose, Insulin, Lipids, Bodyweight

## Abstract

**Background:**

Given the highly debated role of dairy food consumption in modulating biomarkers of metabolic syndrome, this study was conducted to examine the influence of long-term (6 month) dairy consumption on metabolic parameters in healthy volunteers under free-living conditions without energy restriction.

**Methods:**

Twenty-three healthy subjects completed a randomized, crossover trial of 12 months. Participants consumed their habitual diets and were randomly assigned to one of two treatment groups: a high dairy supplemented group instructed to consume 4 servings of dairy per day (HD); or a low dairy supplemented group limited to no more than 2 servings of dairy per day (LD). Baseline, midpoint, and endpoint metabolic responses were examined.

**Results:**

Endpoint measurements of body weight and composition, energy expenditure, blood pressure, blood glucose, and blood lipid and lipoprotein responses did not differ (*p* > 0.05) between the LD and HD groups. HD consumption improved (*p* < 0.05) plasma insulin (-9%) and insulin resistance (-11%, p = 0.03) as estimated by HOMA-IR compared with the LD group.

**Conclusions:**

Study results suggest that high dairy consumption (4 servings/d) may improve insulin resistance without negatively impacting bodyweight or lipid status under free-living conditions.

**Trial registration:**

Trial registration: NCT01761955

## Background

Dairy has been an important component of the human diet, mainly valued for its superior amino acid composition, high protein quality, and a source of calcium [1]. Beyond it’s macronutrient composition, low fat dairy foods contain an array of health promoting bioactive components including whey peptides, conjugated linoleic acid, sphingolipids, oligosaccharides, and imunoglobulins [2]. However, despite this rich bioactive composition, the health effects of dairy are highly disputed [3,4].

Increased dairy consumption has been demonstrated to reduce obesity [5] and modulate metabolic disturbances including hyperinsulinemia [6] and blood pressure [7,8]. Prospective cohort studies suggest that consumption of low fat dairy products is associated with a low incidence of type 2 diabetes compared with low dairy diets [9-12]. A recent meta-analysis of 7 cohort studies by Tong et al. [11] suggests that increased consumption of total and low fat dairy products may reduce the risk of type 2 diabetes by 5 and 10%, respectively. This disease reduction potential may be linked with the insulin sensitizing properties of dairy products as Ruidavets et al. have reported that high consumption of dairy products is associated with a lower probability of insulin resistance (odds ratio 0.67) [13].

Alternatively, it has been suggested that long-term high fat dairy consumption may underlie the pathogenesis of type II diabetes by promoting β-cell apoptosis [14] and increase CVD risk by contributing to hyperlipidemia. These negative health responses may be linked with increased intake of insulin-like growth factors (1 & 2) [3] and saturated fat of dairy foods [15-17]. Inconsistent data from different studies on the health benefits of dairy may be attributed to differences in experimental design (study population, health status, ethnicity, and gender) [18] and differential health effects of specific dairy products [19]. Furthermore, it has been suggested that the health promoting effects of increased dairy consumption, particularly with respect to weight loss, may be associated with an energy restricted diet [20]. Additional long-term randomized controlled trials are needed to resolve the current controversies regarding the effects of increased dairy consumption on risk factors for type 2 diabetes, in addition to other metabolic health responses. Therefore, the aim of the present study was to examine the long-term (6 months) effects of high dairy consumption (4 servings per day) on metabolic parameters in healthy volunteers under free-living conditions without energy restriction or other lifestyle modifications. The primary aim of this study was to observe the effect of dairy consumption on metabolic parameters including blood lipids, glucose, and insulin. Secondary endpoints were body weight and composition, and energy expenditure.

## Methods

The study was conducted at the Clinical Nutrition Research Unit at the Richardson Centre for Functional Foods and Nutraceuticals (RCFFN), University of Manitoba between April 2009 – May 2011. Subjects were recruited from the Winnipeg area via newspaper and radio endorsements, poster advertisements, and flyer distributions.

### Inclusion/exclusion criteria

Male and female subjects (18–75 years of age) were eligible for study participation if they had a BMI between 18.5-35.0 kg/m^2^ and were assessed to be healthy based on a pre-study screening examination including medical, diet, and lifestyle history. Age and BMI inclusion ranges were wide-ranging in anticipation of difficulty in recruiting subjects for a 1-year intervention. Subjects were excluded if pregnant; diagnosed with diabetes or cardiovascular, liver, or renal disease; reported regular use of appetite suppressants or Orlistat (Xenical) or irregular use of other treatments which might interfere with the outcomes of the study (e.g. anti-hypertensives, statins, thyroxine, omega-3 supplements); reported habitual consumption of more than 1 ½ servings of dairy per day; or were unable to consume 4 servings of low fat dairy per day for 6 months due to known allergy or dairy intolerance. To confirm individual health status and eligibility, all subjects underwent a complete physical examination conducted by the study physician.

### Study design and protocol

The study protocol was approved by the Joint Faculty Research Ethics Board (JFREB) at the University of Manitoba. All subjects signed informed consent to participate in the study.

**Figure 1:**
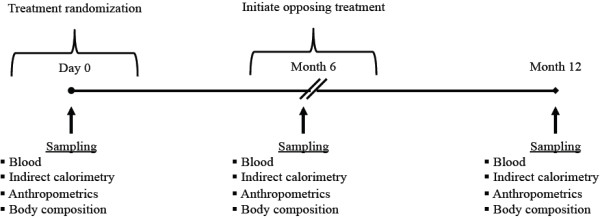
Experimental design followed to examine metabolic response to consumption of low versus high dairy foods.

The study was a randomized, crossover trial of 12 months with no washout period due to the length of each study phase (Figure 1). At the onset of the study, eligible participants (n = 39) were instructed to consume their habitual diets and randomly assigned to one of two treatment groups for 6 months using a random number generator: a high dairy supplemented group instructed to consume 4 servings dairy per day (HD); or a low dairy supplemented group limited to no more than 2 serving dairy per day (LD). At the end of the initial 6-month phase, subjects initially randomized to the HD group were switched to the LD treatment and vise versa for the remainder of the study. The LD and HD serving sizes were based on Canada’s Food Guide, which recommends 2–3 servings of low fat dairy products per day depending on age and sex. However, it has been reported that 65-80% of Canadian adult consume less than 2 servings per day [21]. Subjects assigned to the HD group were regularly provided (every 2 weeks) with standard servings of low fat dairy products (eg. 250 mL milk, 175 g yogurt) for dietary incorporation as indicated in Canada’s Food Guide. Subjects were instructed to consume only those dairy products that were provided by the research staff. During the HD phase, subjects were asked to incorporate dairy products into their diets by substitution so as not to increase their normal energy intake. During the LD phase, subjects were instructed to consume their normal diets but to not exceed more than 2 servings of dairy per day. Nutrient composition of the low fat dairy products is presented in Table 1. Subjects were instructed to maintain their normal diet and level of physical activity for the duration of the study periods. Beyond instructions as to how to incorporate dairy into their daily diet, the subjects received no dietary counseling throughout the study period.

**Table 1:** Macronutrient composition of low fat milk and yogurt products provided to study subjects

**Macronutrient**	**Skim milk**^**1**^	**Yogurt**^**1**^
Serving size	250 mL	100 g
Energy/serving (kJ)	370	180.03
Carbohydrate (g)	12.84	6
Sugar	13.18	6
Fiber	0	0
Protein (g)	8.72	4
Fat (g)	0.21	0.08
Saturates (g)	0.156	0
Trans fat (g)	0.008	0

^1^Provided by Aliments UTLIMA Foods Inc. / Yoplait, Granby, QC.

### Data collection time points

At two separate points during each phase (4 and 6 months of each intervention phase), subjects were asked to complete a food frequency questionnaire (adapted from the National Institutes of Health Diet History Questionnaire), physical activity diary, and 3-day mid-week food record. To monitor compliance, subjects were provided with a logbook and asked to record the number of dairy servings consumed each day. At baseline and the end of each 6-month period, fasting measurements of body weight, blood pressure, DEXA, and indirect calorimetry were obtained. Furthermore, 12-h fasted blood draws were collected for blood biochemistry measurements at baseline, midpoint, and endpoint as outlined in Figure 1. Subject anthropometric and metabolic measurements taken prior to the start of the study are provided in Table 2.

**Table 2:** **Baseline characteristics of study subjects prior to start of study**^**1**^

**Variable**	**Mean ± SD**
General characteristics	
Age (y)	53 ± 12.26 (range 22–72)
Gender (M/F)	5/18
Height (m)	1.65 ± 0.08
Body weight (kg)	85.61 ± 10.30
Body mass index (kg/m2)	31.86 ± 3.01
Waist circumference (cm)	99.76 ± 9.83
Waist:hip	0.84 ± 0.07
Systolic blood pressure	125.34 ± 17.90
Diastolic blood pressure	77.82 ± 14.12
DEXA	
Total body fat (%)	47.46 ± 4.51
Abdominal fat (%)	47.36 ± 8.03
Lean body mass (%)	50.95 ± 4.64
Plasma biochemistry	
Glucose (mmol/L)	5.17 ± 0.68
Insulin (μU/mL)	15.37 ± 2.80
Total cholesterol (mmol/L)	5.73 ± 0.62
LDL-cholesterol (mmol/L)	3.49 ± 0.57
HDL-cholesterol (mmol/L)	1.42 ± 0.37
Triglycerides (mmol/L)	1.79 ± 0.54
ApoB (mg/dL)	85.56 ± 31.88
ApoE (mg/dL)	5.00 ± 1.66
Lp(a) (mg/dL)	52.20 ± 60.58
ApocII (mg/dL)	5.82 ± 2.57
Energy expenditure	
Metabolic rate (kJ/min)	2.80 ± 0.50
Carbohydrate oxidation (g/min)	0.02 ± 0.04
Fat oxidation (g/min)	0.1 ± 0.02

^1^n = 23 for LD; n = 23 for HD.

### Procedures

#### Blood collection and blood pressure

Twelve-hour fasting serum and plasma blood samples were collected at the beginning and end of each study phase. Blood was centrifuged at 3000 *× g* for 20 minutes at 4°C to separate serum and plasma from erythrocytes, which were then stored at -20°C for future analyses. Resting blood pressure and heart rate (average of two measurements) were recorded by automated oscillometry with the subject in supine position.

#### Body composition protocol

Body composition, including percentage of total fat mass (%TFM), TFM and total lean mass, as well as percentage android and gynoid fat, were assessed at the start and end of each study phase using fan beam dual-energy X-ray absorptiometry (LunarProdigy Advance; GE Healthcare, Madison, WI, USA). Body composition data including %TFM, TFM, and total lean mass were determined using Encore 2005 software version 9.30.044 (GE Healthcare).

#### Energy expenditure

At the beginning and end of each study phase, fasting energy expenditure was determined using open circuit indirect calorimetry (Vmax Encore, Summit Technologies Inc, Burlington ON Canada) fitted with a ventilated canopy. The flow sensor and gas sensors were calibrated daily prior to initiating respiratory measurements. The flow sensor was calibrated using a calibration syringe. Gas sensors were automatically calibrated by Vmax Encore software (Cardinal Health, Maple, ON Canada) using two reference gasses with the first containing 16% O_2_, 4% CO_2_ and 80% N_2_ and the second containing 26% O_2_, 0% CO_2_ and 74% N_2_. All indirect calorimetry data was collected using Vmax Encore software. Commencement of pre-menopausal women into the study was planned such that they did not undergo indirect calorimetry measurements during their menstrual cycle. Indirect calorimetry measurements were taken for 30 min. The first 15 min acted as a stabilization period, while data from the latter 15 min was used to determine fasting energy expenditure, fat oxidation and carbohydrate oxidation. The methodologies used to calculate these measures are discussed elsewhere [22]. As subjects were fasting, the constant 0.829 L O_2_/g glycogen was used to calculate carbohydrate oxidation [23,24].

#### Blood biochemistry analyses

Plasma total cholesterol (TC), high-density lipoprotein cholesterol (HDL-C), triglycerides (TG), and glucose were determined by automated enzymatic methods on a Vitros 350 chemistry analyzer (Ortho-Clinical Diagnostics, Markham, Ontario, Canada). LDL-C concentrations were estimated by the difference method using the Friedewald formula. Apolipoproteins were measured by immunoturbidometric assay on a Pentra 400 autoanalyzer according to manufacturer’s instructions (Kamiya Biomedical Company, Seattle, WA, USA; kits: ApoB, KAI-004; ApoE KAI-007; CII, KAI-005; Lp(a), KAI-044). Serum insulin was analysed by ELISA (EZHIASF-14K, Millipore, Billerica, MA). Insulin homeostasis modeling assessment (HOMA) was utilized as an estimate for insulin resistance (IR). HOMA values were calculated using previously outlined methods [25].

#### Statistical analysis

The study was powered based on previously reported lipid responses following 21 weeks of high dairy consumption [26,27]. A sample size of 20 subjects was anticipated to detect a 19% reduction in blood TG (alpha 0.05; 0.80 power). In anticipation of a large dropout rate due to the long study duration, 39 subjects were recruited to begin the trial. Data were analyzed with a linear mixed model ANOVA with subject as a random factor. Baseline measurements of each phase were used as covariates for endpoint measurements. Data were further analyzed as percent change from baseline responses for LD vs. HD phases. Macronutrient intake data collected from mid-week 3-day food records were analyzed with Food processor (Food processor, Salem, OR). All data are presented as means ± SD and statistical significance was set at p < 0.05 for all analyses. Data were analyzed using IBM SPSS software version 19.0 (SPSS, Inc., Chicago, IL, USA).

## Results

**Figure 2:**
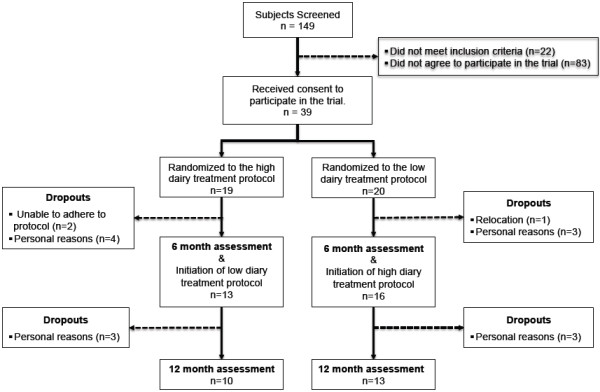
Subject recruitment and progression throughout the study.

One hundred and forty-four individuals initially responded to the recruitment strategy that focused on dairy as a diet intervention for improvement of cardiometabolic risk factors. Of these 144 respondents, 51 individuals were screened and deemed eligible for the trial, and 39 subjects agreed to participate. Of the initial 39 subjects that began the study, 23 completed both study phases (n = 23 for both LD and HD). Subjects provided several reasons for dropping out of the study including an inability to consume the required daily amount of dairy (n = 2), personal family obligations (n = 13), and relocation (n = 1) (Figure 2). Baseline characteristics of dropout subjects were not different (p > 0.05) than those who completed the study (data not shown).

Macronutrient intake during the two periods did not differ (*p* > 0.05) between the LD and HD groups (Table 3). Calcium intake in the HD group tended to be higher (p = 0.10) than the LD group whereas there was no difference (p > 0.05) in vitamin D intake between groups (Table 3).

**Table 3:** **Selected daily nutrient intake estimated from 3-day mid-week food records**^**1**^

**Item**	**Low dairy**^**2**^	**High dairy**^**2**^
Total calories	2396 ± 430	2268 ± 502
Carbohydrate (% energy)	51.08 ± 6.93	54.13 ± 6.71
Fat (% energy)	34.06 ± 5.72	30.07 ± 4.75
Protein (% energy)	15.98 ± 3.29	15.81 ± 1.74
Total Carbohydrates (g)	299.66 ± 139.38	307.65 ± 83.07
Monosaccharides (g)	13.54 ± 10.11	10.87 ± 8.03
Disaccharides (g)	13.48 ± 4.76	8.92 ± 3.15
Total dietary fiber (g)	25.58 ± 9.04	23.79 ± 8.41
Total Fat (g)	94.55 ± 38.12	76.09 ± 22.08
Saturated fat (g)	26.72 ± 9.44	24.1 ± 8.52
Monounsaturated fat (g)	19.55 ± 9.97	15.06 ± 7.96
Polyunsaturated fat (g)	9.55 ± 3.37	8.74 ± 3.09
Cholesterol (mg)	273.29 ± 96.62	217.87 ± 77.03
Total Protein (g)	94.32 ± 42.83	88.14 ± 31.16
Vitamin D (μg)	4.77 ± 1.68	6.17 ± 2.18
Calcium (mg)	1024.31 ± 347.77	1222 ± 163.15

^1^Estimated from 3-day food records taken at 4 and 6 months of each intervention phase.

^2^Mean ± SD; n = 23.

Endpoint and percent change adjusted whole-body and metabolic responses between the LD and HD phases are presented in Table 4. Although no difference (*p* > 0.05) was observed in blood glucose between the LD and HD groups, HD consumption reduced (*p* < 0.05) plasma insulin and improved (*p* < 0.05) insulin resistance as estimated by HOMA-IR, (endpoint and percent change Table 4). Endpoint and percent change variables in body weight and body composition, energy expenditure, blood pressure, and blood lipid and lipoprotein responses did not differ (*p* > 0.05) between the LD and HD phases.

**Table 4:** Metabolic parameters at the end of the 6 month low and high dairy phases

**Variable**	**Endpoint**	**% change**	**Endpoint**	**% change**
	**LD**^**1**^	**LD**^**2**^	**HD**^**1**^	**HD**^**2**^
Body weight (kg)	86.2 ± 8.6	2.1 ± 1.2	87.0 ± 8.2	2.4 ± 1.3
Waist circumference (cm)	100.7 ± 13.3	1.0 ± 2.3	98.2 ± 7.3	1.2 ± 2.2
Total body fat (%)	45.4 ± 9.4	1.9 ± 2.1	45.0 ± 8.6	2.7 ± 2.3
Abdominal fat (%)	44.8 ± 9.02	2.1 ± 2.62	44.9 ± 7.82	1.2 ± 1.53
Systolic blood pressure (mm/Hg)	124.1 ± 16.2	2.5 ± 3.2	121.5 ± 14.6	4.8 ± 2.5
Diastolic blood pressure (mm/Hg)	78.5 ± 16.0	0.7 ± 2.6	76.6 ± 9.4	2.3 ± 2.6
Glucose (mmol/L)	5.2 ± 0.7	3.8 ± 1.5	5.2 ± 0.7	2.1 ± 1.9
Insulin (μU/mL)	16.2 ± 3.7	10.0 ± 3.2	14.8 ± 2.4^*^	2.5 ± 2.2^**¥**^
HOMA-IR	3.8 ± 1.3	16.5 ± 4.6	3.4 ± 0.9^*^	5.1 ± 2.8^**¥**^
Total cholesterol (mmol/L)	5.4 ± 0.9	1.1 ± 3.1	5.6 ± 0.8	3.1 ± 2.4
LDL-cholesterol (mmol/L)	3.3 ± 0.8	-1.2 ± 2.1	3.4 ± 0.8	3.9 ± 5.1
HDL-cholesterol (mmol/L)	1.4 ± 0.3	4.6 ± 2.3	1.4 ± 0.4	8.4 ± 3.4
Triglycerides (mmol/L)	1.8 ± 0.7	-0.05 ± 8.7	1.7 ± 0.6	-3.8 ± 10.1
ApoB (mg/dl)	90.7 ± 20.1	2.1 ± 1.8	92.5 ± 19.6	3.2 ± 4.5
ApoE (mg/dl)	5.4 ± 0.5	-2.5 ± 1.1	5.3 ± 0.8	-4.8 ± 2.6
Lp(a) (mg/dl)	37.3 ± 37.9	-9.4 ± 6.2	48.1 ± 13.1	-0.05 ± 7.4
ApoCII (mg/dl)	6.5 ± 1.7	10.1 ± 3.9	6.2 ± 1.9	2.6 ± 3.9
Metabolic rate (kJ/min)	2.8 ± 0.7	1.0 ± 3.9	2.7 ± 0.8	4.9 ± 4.3
Carbohydrate oxidation (g/min)	0.01 ± 0.04	3.6 ± 1.7	0.03 ± 0.05	6.8 ± 3.2
Fat oxidation (g/min)	0.07 ± 0.02	3.8 ± 0.02	0.06 ± 0.02	9.6 ± 10.9

^1^Endpoint values adjusted for baseline levels within period; mean ± SD; n = 23 LD; n = 23 HD.

^2^% change from baseline values were calculated using phase 1, d-1.

*P < 0.05 compared with endpoint LD; ^**¥**^P < 0.05 compared with % change from baseline LD.

## Discussion

The major finding of this study is that consumption of 4 servings/d of low-fat dairy milk and yogurt products under free-living conditions for 6 months reduced fasting plasma insulin (9%) and improved insulin resistance (11%) in overweight and obese adults. Although the long-term clinical implications of these results are unclear, fasting insulin and HOMA-IR have been shown to be sensitive predictive markers of diabetes, ischemic stroke, and coronary heart disease risk in the general population as well as in at-risk subjects [28-32]. Previous data from the Verona Diabetes Complications Study suggest that every 1-unit increase in HOMA-IR is associated with an odds ratio of CVD incidence of 1.56 in Type II diabetics [33]. This improvement in insulin resistance may be protective in this population of overweight and obese individuals.

Our results are supported by dairy intervention studies under both controlled feeding and free-living conditions. Compared to a low dairy diet (<0.5 servings/d), a 12-week study by Stancliffe et al. (2011) reported reductions in fasting insulin and insulin resistance in a similar population of overweight and obese subjects consuming dairy as milk and yogurt (3.5 serving/d) [6]. Similar results were recently reported by Nikooyeh et al. (2011) [34] in type 2 diabetics consuming 250 mL/day of plain, vitamin D-fortified, or a vitamin D + calcium-fortified yogurt drink. Moreover, results from a recent meta-analysis and previous observational and prospective cohort studies support long-term dairy consumption in lowering the incidence of type II diabetes and metabolic syndrome [11,35-37].

Alternatively, a recently published 12-month cross-over study examining the cardiometabolic health responses to increased dairy consumption by Crichton et al. [38] did not observe an insulin sensitizing response to HD vs. LD intake. This study was similar to the present study in terms of design (crossover), length (6 month), dairy servings (HD, 4 servings/d vs. LD, ≤ 1 servings/d), and study population (overweight and obese). However, while our study limited dairy products to low fat milk and yogurt, the study design by Crichton et al. was more inclusive by incorporating low fat flavored milk and yogurt, vanilla custard, and a limited amount (no more than 7 servings/week) of cheese (sliced, cottage, ricotta, cream), ice cream, butter, and margarine. We deliberately utilized milk and yogurt as treatments because these dairy products have reasonably long expiration dates, are easily portable, and can be readily incorporated into the diet. Depending on ingredient composition, formulation, and manufacturing process, dairy products have a broad range of textural and physiochemical properties. These differences greatly influence the bioavailability of dairy-derived nutrients and bioactive compounds and may ultimately affect the magnitude and direction of health responses following their consumption. Although our results suggest that consumption of dairy foods in the form of milk and yogurt may have beneficial cardiometabolic effects, it is difficult to speculate if similar responses would be observed in response to the consumption of other dairy foods. Ivey et al. (2011) [19] recently reported that, compared to milk and cheese, yogurt consumption decreased carotid artery intima-media thickness in elderly women. Thus, studies designed to specifically examine health responses to different whole dairy foods and isolated dairy food bioactive components are clearly warranted.

Numerous milk-derived bioactive compounds may contribute to the insulin-sensitizing effects of dairy including calcium, vitamin D, whole whey protein, and fractionated peptides [39,40]. Vitamin D is well recognized in sensitizing insulin responses through multiple purported mechanisms which include regulation of insulin receptor expression and stimulation of insulin release by pancreatic β-cells [41,42]. Although both milk and yogurt products used in the present study were vitamin D fortified as stipulated under Canadian federal law, we observed no difference in vitamin D intake between the LD and HD phases, suggesting that the insulin sensitizing response to increased dairy intake was independent of vitamin D consumption. However, as this study was designed as free-living and assessed nutrient intake using food records, it is subject to design limitations including poor dietary recall [43,44]. Limitations of dietary recall during the 3-day food record may also be responsible for the tendency (*p* = 0.1) observed in calcium intake between the HD (1222 mg) and LD (1024 mg) groups.

Given that the present study was designed to examine the combined health response to low-fat milk and yogurt whole foods, we cannot partition the effects of the specific dairy foods or identify the particular bioactive component(s) that may be responsible for the protective effects against increased insulin resistance. The vast majority of studies examining the influence of dairy consumption on biomarkers of metabolic syndrome have been conducted with whole dairy foods with very few studies designed to partition the specific health effects of isolated dairy components. One notable exception is a recent study by Palacios et al. (2011) [26] who reported no change in body composition or serum lipids in obese subjects instructed to consume low calorie diets providing ~1200 mg/d calcium from dairy or calcium supplements.

Concerns have been raised that high dairy consumption may be associated with an increased risk of dyslipidemia and obesity [3]. However, the results of this study lend further support to a growing clinical trial database suggesting that low fat dairy foods can be incorporated into diets without adversely raising blood lipids [38,45-47]. Similarly, there is no clear consensus of the long-term effects of dairy consumption on body weight and body composition, with reports of both increased [48], decreased [49-51], and neutral [52,53] body weight changes following dairy consumption. A recent systematic literature review by Kratz et al. examining 16 studies [54] found little support for the hypothesis that increased dairy fat or high fat dairy food consumption contributes to increased adiposity. Similarly, a meta-analysis from Chen et al. [55] using 29 RCT recently examined the effects of dairy consumption on body weight and body fat mass. Their results suggest that dairy consumption may not be associated with weight gain, but may have a modest effect in facilitating weight loss in energy restricted and short-term (<1 yr) intervention studies. Results from the present 6 month study demonstrate that, under free-living conditions and without energy restriction, long-term low-fat dairy consumption does not alter body weight, body composition or energy expenditure. These results further suggest that the insulin sensitizing effects of dairy consumption are not related to body fat loss or factors that modulate energy metabolism.

There are several limitations to this study. First, it proved difficult to sustain volunteer interest over such a long trial duration and only 23 subjects of the original 39 fully completed each of the study phases (41% dropout rate); however, we were able to retain our desired target of 20 subjects. Interestingly, the previously discussed study by Crichton et al. [38] with a similar design to ours also had a high drop-out rate (49%), which suggests that future studies involving high dairy consumption may benefit from a parallel arm design. Until a similar study is repeated with a larger study population, our results should be interpreted with caution. Second, although volunteers were given log books to record their daily dairy intake, these records were not reviewed by the study staff until the end of the study, at which point it was determined that they were incomplete. Therefore, the lack of a compliance evaluation and actual dairy intakes are a major limitation of the current study. Third, subjects were provided with dairy products during the HD phase but were not provided with dairy-free foods of comparable macronutrient composition during the LD phase. This could have resulted in differences in food intake and/or macronutrient balance between the two phases beyond the sensitivity of the dietary analysis conducted.

## Conclusions

In conclusion, the current study suggests that under free-living conditions, consumption of 4 servings/d of low-fat dairy milk and yogurt products for 6 months may improve insulin resistance without negatively impacting bodyweight or lipid status. These results require verification from additional long-term studies with appropriate measures to increase subject retention. Future studies should also be designed to further examine the metabolic effects of specific dairy products and/or dairy-derived bioactive components.

## Abbreviations

HD: High dairy; LD: Low dairy

## Competing interests

The authors declare that they have no competing interests. This study was supported in part by a Science & Technology International Collaboration (STIC) grant from the Manitoba Department of Innovation, Energy and Mines.

## Authors’ contributions

TCR and CPFM analyzed the data and wrote the initial draft manuscript; HM coordinated the intervention trial and sample analysis; RWB conducted the lipid and lipoprotein analysis; CBR designed and coordinated the study. All authors read and approved the final manuscript.
